# Entrenched Inequalities? Class, Gender and Ethnic Differences in Educational and Occupational Attainment in England

**DOI:** 10.3389/fsoc.2020.601035

**Published:** 2021-01-28

**Authors:** Yaojun Li

**Affiliations:** Department of Sociology and Cathie Marsh Institute for Social Research, School of Social Sciences, Manchester University, Manchester, United Kingdom

**Keywords:** class, ethnicity, gender, educational attainment, labor market position, England

## Abstract

Research in social stratification tends to focus on class differences in educational and occupational attainment, with particular attention to primary and secondary effects in the former, and class reproduction in the latter, domain. Research in ethnic studies tends to focus, however, on ethnic penalty or premium. Many studies have been conducted in each tradition on specific issues but little research is available that examines class, gender and ethnic effects simultaneously or in tandem with contextual effects, let alone on the whole trajectory from compulsory schooling, through further and higher education, to labor market position. Using data from the Longitudinal Study of Young People in England, this paper shows pronounced class differences but remarkable gender progress in each of the educational domains. With regard to ethnicity, people from minority ethnic heritages had lower GCSE scores due to poorer family conditions but achieved higher transition rates to A-Level study, higher university enrollment and, for some groups, greater attendance at elite universities, resulting in an overall higher rate of degree-level attainment than did whites. One might expect members of ethnic minority backgrounds to fare equally well in their earlier careers in the labor market, but only to find them more vulnerable to unemployment, less likely to have earnings, and more disadvantaged in terms of disposable incomes.

## Introduction

The aim of this paper is to study the educational and occupational achievement of members of second-generation ethnic minority groups in England, whether they are subject to similar class effects as those from the majority group, and whether there are specific ethnic penalties in their educational trajectory from compulsory schooling to higher education and furthermore in their early careers in the labor market. Sociologists have conducted many studies on how family origins affect children’s educational and occupational attainment in Britain. Most of the studies are focused on educational attainment in compulsory schooling and progression to A-Level study given the prior academic performance. Yet, little research is available that combines insights from both social mobility and ethnic studies traditions to examine the entire educational trajectories from compulsory schooling through A-Level studies to higher education, and furthermore into the labour-market position after completion of education, and to interrogate the underlying socio-economic-cultural factors at the individual and community levels in terms of parental class, gender and ethnicity on the one hand, and school-level deprivation and diversity on the other, that shape the trajectories. This paper seeks to make a contribution to knowledge in this respect.

The paper is structured as follows. In the next section, we give a brief account of the sociological analyses on educational and occupational attainment, with particular attention to research on primary and secondary effects, and on ethnic penalty and premia. We show that while many studies have examined the class effects in broad terms on the transition to A-Level studies, no research is currently available that links family class, gender and ethnicity, and also contextual influences to pupils’ performances and transitions in the entire educational journey and moves further afield into labour-market positions. After that, we present data and analyses. The paper will conclude with some discussion.

## Literature Review

Sociologists concerned with social inequality have conducted much research on educational and social mobility. They wish to find out how family condition in terms of parental class, education and income either singularly or in combination with other ascribed characteristics such as gender and ethnicity affects people’s opportunities and outcomes in educational and occupational attainment. Yet, as Li and Heath (2016) point out, whilst sharing the same goal of investigating social inequality, mainstream sociologists and ethnic studies scholars have largely traveled on separate tracks, with the former concerned with class effects and the latter with ethnic penalties (and, more recently, ethnic premia, see Heath and Brinbaum, 2014).

As education plays a pivotal role in increasing people’s human capital, broadening intellectual horizons and serving as a passport to the labor market, it is a major arena of class competition, academic debate and policy-making. The classical modernization theory proposes that with economic development and growing government provision of educational services, achievement between children from different social class origins will become increasingly equal and family influences will gradually pale into insignificance. The increasing influx of visible ethnic minority groups into Britain has posed a serious challenge to the theory: can it explain the process of educational stratification for immigrants’ children equally well as it does for the majority population? Here the first task is to test whether the theory can really explain the patterns and trends of educational attainment for the mainstream (majority) population, and the second task is to see how well it explains the educational attainment for the second-generation ethnic minority groups. How big an effect does origin class have on children’s attainment? Do class differences in children’s educational attainment stay constant or become stronger or weaker over time with greater government provision of educational services? Does the class position of immigrant families play an equally protective role in their children’s education as that of the majority families? Do ethnic minority children from advantaged class backgrounds suffer a “perverse fluidity” and experience excessive downward mobility as earlier studies found for African Americans in the United States (Duncan, 1968; Hout, 1984)? Or do immigrant children show greater aspiration, resilience and determination for more education despite family disadvantages?

In a landmark study on social stratification of education, Halsey et al. (1980: 184) show pronounced class differences in education and increasing differentials at higher levels of educational attainment. For instance, 71.9% of the men from professional and managerial “service-class” origins attended selective secondary schools as compared with only 23.7% of working-class sons, at a disparity ratio of 3.0:1. The ratios became 4.9:1, 9.6:1 and 11.2:1 at O-Level, A-Level and University attendance respectively.

Do class differences in educational attainment stay constant or do they show signs of aggravation or amelioration? Breen et al. (2009) used the pooled data from the General Household Survey (1973–1992) to study educational stratification in Britain in comparison with seven other industrial societies. Using a semi-cohort approach, the authors showed that class differences in educational attainment were being consistently reduced for men from successive birth cohorts from 1908–24 to 1955–64, and this result obtained whether one used country-specific data or with class and educational variables standardized across countries. Similar patterns of declining social inequality in educational attainment was found for women (Breen et al., 2010), lending support to the modernization theory. The authors attribute this to the reduction in family resources and the government provision of educational services after the end of the Second World War, yet this is contrary to economists’ findings of declining social mobility in education (Blanden et al., 2005).^1^

Why do children from different classes have different educational outcomes? One theory is that their families have differential possession of resources. Bourdieu (1986) holds that middle-class families possess cultural, social and economic capitals beyond the reach of working-class families, and that it is differences in family resources that will engender differences in educational outcomes. For instance, middle-class families tend to use their superior resources to help their children’s education by creating a pro-learning family environment, practising “concerted cultivation” (Lareau, 2003), moving to more expensive catchment areas where good-quality state schools are located or sending the children to private schools. Perhaps most importantly, according to Bourdieu, middle-class families equip their children with a habitus which enables them to “move in their world as a fish in water” whereas the anti-learning attitude of working-class children makes them feel like “fish out of water” in educational environments (Bourdieu, 1990: 108).

Bourdieu’s cultural capital theory is challenged by Goldthorpe (2007a) who finds it inherently flawed, that is, incompatible with the observed facts. In Britain as in other developed countries, working-class children have steadily increased their attendance beyond compulsory education in the last few decades. If there is a working-class habitus which instils an anti-learning attitude in them and which makes them feel like a fish out of water in school, why would their attendance rates have increased so much? The very fact of increasing attendance suggests that working-class children are not as anti-learning as the habitus theory would imply, but have an eagerness for more advanced learning if their family resources would allow them to. In an effort to provide an alternative and more viable explanation, Goldthorpe developed the “rational action theory” (RAT), also called “relative risk aversion” (RRA) theory (Breen and Goldthorpe, 1997, 2001; Goldthorpe, 2000, 2007b, 2014; see also; Kahneman, 2011) to explain both the increasing working-class uptake of education at the absolute level and the constant differential with the middle class uptake at the relative level. Key in the RRA thesis is the proposition that parents in all social positions would wish their children to do at least as well as they themselves have done in terms of educational and occupational attainment and to try to avoid downward mobility. When children are faced with the need to make decisions as to whether or not to proceed to a more advanced level of study or to enter the labor market at the end of compulsory schooling, they will consult with their parents. The outcome of such consultation tends to be that working-class children with more limited socio-cultural-economic resources will usually make “realistically feasible” decisions (called “strategy from below”) whereas middle-class children, backed by superior resources, will more often than not make more ambitious decisions, even when they have similar or even lower levels of academic performance as compared with working-class children (called “strategy from above”). This tendency to exercise caution (risk aversion) in the case of working-class children and to embrace challenge (risk venture) in the case of middle-class children underlies the distinction between the primary and the secondary effects, a distinction made by Boudon (1974). The primary effects may be of a genetic or socio-economic-cultural kind, and refer to levels of academic performance that are actually achieved by children from different class origins. It is usually the case that students from more advantaged backgrounds have higher levels of performance than do those from more disadvantaged backgrounds. The secondary effects refer, however, to the different choices that children of different class origins will tend to make in consultation with their parents at critical junctions on their educational journey from compulsory (GCSE) to post-compulsory work such as transition to A-Level and, furthermore, to undergraduate and post-graduate studies in England. Both the “realistically feasible” choices and the more “ambitious” choices are deemed rational by the actors given the circumstances in which they find themselves.

Goldthorpe and his colleagues have made several efforts to test the thesis of primary vs. secondary effects. Using the National Child Development Study of 1958 when the respondents turned 16 in 1974, and two Youth Cohort Study (YCS) datasets where the respondents were also aged 16 (in 1987 and 2002), they find that people from professional-managerial (“service-class”) families have higher scores in English and mathematics examinations than do working-class students in each of the three cohorts, which is as expected. Yet, they also find that, even at similar levels of academic performance, students from service-class families have a higher likelihood of transition into A-Level work than do working-class students, by around 15 to 20 percentage points; that there is little change over time in the class differentials from 1974 to 2002; and that secondary effects account for around one quarter to one half of the class differentials in educational attainment (Erikson et al., 2005; Jackson et al., 2007; Goldthorpe and Jackson 2008). These findings lend powerful support to the rational action theory. Yet it is also the case while these are among the best research findings in this area, they only differentiate three broad origin classes without taking gender or ethnicity into consideration. Jackson (2012) tried to improve upon the situation by pooling three YCS datasets together (when students turned 16 in 1998, 2000 and 2002) and analyzing the transition rates to A-level and to university studies between different ethnic groups. The primary effects are measured by standardized scores in the public examinations of mathematics and English at GCSE, and of A-level grades, and the secondary effects by class-based transition rates given prior levels of performance. She found that most ethnic groups had lower test scores but higher transition rates than did the white majority group, which she interpreted as evidence of significant disadvantages in the primary effects but significant advantages in the secondary effects. Jackson holds that the former runs counter to claims of positive selection (ethnic premium) as proposed by scholars in prior research but the latter indicates a defensive strategy against possible discrimination at the hands of employers. Jackson’s view of the higher transition rates by ethnic minority groups as a defensive strategy makes sense in light of the systematic findings on barriers faced by ethnic minority groups in the British labor market (Berthoud, 2000; Li and Heath, 2008; Li and Heath, 2018; Heath and Di Stasio, 2019) although to term such “defensive strategies” as an advantage seems debatable.

While early studies may have a reasonable excuse to ignore the issue of ethnicity on grounds of data limitation, the rapid increase of the visible ethnic minority composition in the population indicates that any continued adoption of an ethnic-blind approach is no longer viable. Given this, researchers have paid increasing attention to second (or multiple) generation ethnic experiences in education, access to employment and career advancement (Heath and Brinbaum, 2014; Li, 2018b; Lessard-Phillips and Li, 2017). Yet it has been difficult to accommodate the conventional class analysis approach with the ethnic studies approach. For instance, one may aptly term middle-class children’s greater educational ambition a resource-based “advantage”, because middle-class families do have superior resources of various kinds relative to working-class families, but in what sense can we call the higher transition rates by poverty-ridden ethnic minority students an “advantage”? Scholars have made a few suggestions as to why ethnic minority children who come from poorer families and who achieve lower test scores at the stage of compulsory schooling should exhibit higher transition rates to further and higher education, and posited different theses such as “positive selection” (Borjas, 1987; Feliciano, 2005; Ichou, 2014), “consonant acculturation” (Portes and Zhou, 1993), or “reinvigorated aspiration” (Li, 2018a). The positive selection thesis holds that visible ethnic minority immigrants from far-away countries (rather than from nearby countries such as the “guest workers” who moved from Turkey to West Germany after the Second World War) are not a random selection of the population in their country of origin but have exceptional qualities in terms of aspiration, ambition, determination, perseverance and resilience.^2^ The first generation arriving in the receiving country will often meet with multiple handicaps due to a lack of economic capital, disrupted social capital, insufficient cultural and human capital (such as ignorance of the local labor market, low levels of education, possession of foreign qualifications unrecognized by the employers, and poor English) and other factors, and will tend to find themselves in poorly-paid jobs shunned by the mainstream population. But they are determined to survive and thrive, and will pass on their ambition, aspiration, determination and other positive qualities to their children. This thesis sounds attractive but does not explain why there is so much variation among different second-generation ethnic minority groups whose parents came from countries of similar distances to Britain. The segmented assimilation theory (Portes et al. 2009), which proposes three modes of assimilation (consonant, selective and dissonant acculturations), is designed to explain the variation among the different groupings. The most successful group will, according to the theory, adopt the “consonant acculturation” strategy where professional parents and their children will learn the language and culture together and the children will obtain elite middle-class positions upon entry into the labor market, achieving full integration. The second group who adopt the “selective acculturation” strategy will be economically successful but will choose to preserve their unique cultural traditions. The third group with “dissonant acculturation” will join the ranks of the underclass. This theory sounds elegant, but does not stand rigorous empirical test, as the great majority of second-generation children do not fit neatly into any of the modes (Waters et al., 2010). The thesis of reinvigorated aspiration as posited by Li (2018a) assumes that the second-generation, growing up in poor families and poor communities, will have a good understanding accrued from lived/perceived experience and parental communications that, as members of ethnic minority heritages, they are likely to experience disadvantage and discrimination in the labor market, at all processes of job application, interviewing, and gaining promotions in the career life, and therefore have to aim higher now so as not to fall too low in future (see also Carmichael and Woods, 2000; Connor et al., 2004; Modood, 2005; Heath and Li, 2008; Wood et al., 2009; Rafferty et al., 2012; Zwysen and Longhi, 2018). At the core of this thesis is the “signaling” theory (Spence, 1973; Weiss, 1995) which assumes that competitors perceived to be in weaker positions tend to give stronger signals to avoid being ignored and to gain adequate recognition. Previous work applied the idea to analysis of degree-level attainment by the second-generation ethnic minority members in the United Kingdom but the thesis needs further and more rigorous test from the educational trajectory at different junctures to the labor market position in the different spheres to demonstrate its viability. The present analysis is devoted to this task.

To sum up, there has been much research on educational attainment in the United Kingdom but existing work is mostly limited to class effects on performance and transition to A-Level studies. Only a few studies extend to transition to university enrolment. No research has linked the family origin (including class and education), gender and ethnic effects on children’s educational and career trajectories in one go whilst at the same time controlling for other socio-economic factors at the individual and contextual levels. With regard to the last point, we may note that most mobility studies adopt an individualistic approach, yet it is well known that contextual effects play an important role in children’s education, a role keenly appreciated by parents and government decision-makers. Middle-class parents try to buy houses in catchment areas with good schools. Government offices have launched various widening-participation programs to help improve the life chances of children in deprived areas. Yet government analyses tend to focus on indicators of local-area deprivation without looking at parental socio-economic conditions (Social Mobility and Child Poverty Commission, 2015; Social Mobility and Child Poverty Commission, 2016; see also Friedman and Macmillan, 2017) just as academics tend to focus on individual attributes without taking considering contextual effects. Thus academic and government research efforts rely on different data sources and have not been able to form a meaningful dialogue, with the former being susceptible to the “atomistic” fallacy and the latter to the “ecological” fallacy (Robinson, 1951; Li et al., 2005). The present analysis is fortunate in being able to draw data from both personal and contextual perspectives and we hope to ameliorate the situation by including not only respondents’ and their families’ demographic and socio-cultural attributes that have been demonstrated to have an important bearing on primary and secondary effects, but also school-level indicators of family poverty and ethnic diversity. The former refers to the proportion of students being eligible to means-tested free school mean (FSM) and the latter to the Herfindahl index of ethnic diversity in each of the schools that took part in the survey. With these factors in mind, the present study seeks to address the following research questions:

• How do different ethnic groups perform in their GCSE studies as compared with white children and among one another, given their parental class, education, family composition and other socio-economic, including contextual, circumstances?
• How do the different ethnic groups differ in their transitional probabilities to A-Level studies, and to university (including Russell-Group) enrolments?
• ethnic minority children have the same returns to education in the labor market as do their white peers?

## Data and Methods

To address the foregoing questions, this study will use the Longitudinal Study of Young People in England (LSYPE1), also called Nest Steps (NS). The survey represents all young people aged 14 and resident in England attending maintained schools, independent schools and pupil referral units (PRU) in February 2004. It adopted a stratified, multi-stage, and random sampling design with oversamples of the major ethnic minority groups to provide sufficient ethnic samples for statistical analysis. 838 maintained schools, 52 independent schools and two PRUs were sampled. It follows their lives through seven waves annually until 2010, and then again when they were aged 25 in 2015. The initial sample size was 15,770 but at wave 4, a boost sample of 352 respondents was added, with a total size of 16,122. As with other cohort and panel studies, the NS has suffered sample attritions, with only 7,707 respondents being found in Wave 8 (age 25). The NS data can be linked with the National Pupil Database (NPD), which contains information on pupils’ examination results at each key stage, schools and colleges attended, eligibility for legally-defined and means-tested free school meal (FSM), school-level characteristics such as proportion eligible for FSM and proportions of pupils from each of the main ethnic minority groups. The data thus contain a wealth of information at the individual and school levels enabling researchers to make a detailed analysis of the primary and the secondary effects at different stages of educational career, and of the labor market position in their early working careers.

With regard to parental socio-economic position, class in terms of National Statistics Socio-economic Classification^3^ (NSSeC) and education in the form of highest level of qualification will be used with the dominance approach adopted (Erikson, 1984; Li and Devine, 2011), namely, the higher position from father or mother. For single-parent families, his or her class and education will be used as family position. As Ilie et al. (2017) show, parental class and education have better predictive power than family income. Siddiqui et al. (2019: 82) also argue that as over half (58%) of the respondents in the survey had missing data on family income, any attempt to use existing variables to impute missing income would make subsequent analyses of income effects blighted. At a theoretical level, class, as Goldthorpe and McKnight (2006) hold, serves as a better indicator of permanent household income than wages or salaries on grounds of economic security, income stability and future prospect.

Other explanatory variables at individual and contextual levels include family composition, eligibility to free school meal, nativity, school-level deprivation in the form of proportions of students eligible for free school meal, and school-level ethnic diversity as indicated by proportions of students belonging to each of the main ethnic groups. A Herfindahl index was created on ethnic diversity for each school.

We use several outcome variables. The first of these pertains to GCSE test results taken at the end of compulsory schooling. Pupils usually take eight GCSE subjects in England. Some schools also offer students the optional short-course GCSEs which contain roughly half the learning material and count as half a GCSE. A summary score was created with A* = 8, A = 7, B = 6, C = 5, D = 4, E = 3, F = 2 and G = 1 for full GCSEs and A* = 4, A = 3.5, B = 3, C = 2.5, D = 2, E = 1.5, F = 1 and G = 0.5 for half GCSEs. The scores range from 0 to 111; with a mean score of 39.7 with standard deviation 20.7. For some of the analysis, the scores will be standardized with a mean of zero and standard deviation of unity. The other outcome variables pertain to transition rates to A-Level studies at the end of GCSE, and to university enrolment at the end of A-Level study or to elite Russell Group university attendance, and labour-market position including employment status, class position, gross weekly pay among the employed, and the ‘continuous weekly income’ for all respondents at wave 8. The analysis of both kinds of income are necessary as nearly a third of the young adults were workless, including unemployment (5.7%), full-time students (5.0%), looking after home (4.7), sick or disabled (1.7%) or inactivity for other reasons. Analyzing the “continued weekly income” from the perspectives of family class, gender and ethnicity is also important in addition to that of labour market earnings as it will allow us to see how the different social groups are being treated at the societal level. Statistical methods will be adopted as appropriate for the task at hand.

## Analysis

The analysis in this section will focus on the respondent’s educational and early career trajectories from ages 16 to 25. As earlier noted, we shall first analyze ethno-class differences in educational achievement before moving to occupational attainment. We examine GCSE scores at age 16, transition rates to A-Level and to university studies (including attendance at Russell Group universities). In the second part, we shall look at the employment situation and incomes.

### Educational Attainment in Compulsory Schooling

The data in Table 1 show an overall view of the class, ethnic and gender differences in GCSE scores and probabilities of progression to A-Level, university and elite university studies.

**TABLE 1:** Descriptive analysis of GCSE score, transition rate (%) into A-Level, university and Russel-Group (RG) university work by parental class, ethnicity and sex.

	GCSE score	% To A-Level	% To university	% To RG university
Parental class				
Higher salariat	55.0	82.2	67.1	26.2
Lower salariat	46.1	69.6	49.5	11.5
Intermediate	39.9	59.6	36.8	7.1
Own account	37.5	58.6	35.3	5.7
Lower technical	32.0	46.6	25.7	3.0
Semi routine	29.6	46.6	23.1	3.3
Routine	23.9	41.4	18.4	2.1
Ethnicity				
White	39.7	60.2	37.8	9.6
B Caribbean	31.8	53.8	39.4	2.4
B African	36.6	70.1	60.9	8.1
Indian	44.9	78.9	73.9	12.7
Pakistani	34.8	68.9	49.2	7.1
Bangladeshi	34.7	65.8	52.9	8.0
Chinese	59.5	91.0	96.8	29.3
Mixed	40.3	61.0	41.9	10.4
Other	43.5	72.9	52.5	11.0
Sex				
Male	37.3	58.1	36.5	8.9
Female	42.1	65.3	44.1	10.9
(All)	39.7	61.5	40.4	9.7
(Approximate N)	15,755	10,355	8,476	8,476

Notes: Weighted analysis and unweighted Ns. The weights are taken from waves 4, 6 and 7 respectively (the same below). The sample sizes become smaller over the waves but remain sufficient for analysis. Take GCSE for example. The number of respondents from higher salariat to routine origins are 1,697, 4,568, 1,430, 1,594, 1,388, 2056 and 2,350; for ethnicity, the numbers from white to other are 10,330, 703, 748, 1,010, 945, 728, 26, 822 and 417; and for gender, the numbers for men and women are 7,832 and 7,571 respectively.

Source: The Longitudinal Study of Young People in England (LSYPE) (the same below).

With respect to class effects, we find pronounced differences with clear gradients in each of the four domains under discussion. As noted above, the mean GCSE score for the sample is around 40 but we see that people from higher salariat (professional and managerial) families had a mean score of 55 whereas those from routine manual families only had a mean score of 24, with a difference of 31 points. The class differentials increased when we look at the transition rates to A-Level and to university studies, with the differences between the higher salariat and routine students being 41 percentage points in the former and 59 points in the latter regard. And with respect to access to the more prestigious Russell Group universities, as shown under the last column, the class differences are also striking, with over a quarter (26%) of the higher salariat children studying in Russell Group universities in contrast with a meagre two percent for those from routine families.^4^

The middle part of the table shows the data on ethnic differences. As white students comprise an overwhelming majority in the sample (86%), their attainment level closely represents the mean performance in each of the four aspects. We see clear and striking differences both between white and ethnic minority students, and among the ethnic minority groupings. In each of the aspects, Chinese students showed themselves as the highest performers, followed by Indians, in clear contrast with Black Caribbeans. Children from Black African, Pakistani, Bangladeshi families had the lowest GCSE scores but higher transition rates to A-Level and university than white students.

The data on gender differences show no female disadvantage. If anything, girls outperformed boys at each stage. The data on university enrollment echo the historical profile Heath et al., 2018a: 68, **Figure 4.2**) which shows men as having a lead over women in access to higher education from the mid-1950s to mid-1990s but since then, women have caught up with and increasingly surpassed men.

The intriguing question is why students in ethnic minority groups underperform in GCSE examinations but make “bold choices” at transitions to further and higher education. If the most important determinant of academic performance and subsequent choice concerns the “class-lined inequalities of condition” (Goldthorpe and Mills, 2004: 223), it is understandable that ethnic minority students who come from poorer families will have lower performance. But if the secondary effects are also reliant, and even more so than the primary effects, on family resources as the “relative risk aversion” thesis would argue, why would the poorer and worse-performing ethnic minority students make even bolder choices than their more affluent and better-performing white peers rather than take a “realistically-feasible” strategy as the RRA thesis would predict? In other words, if family poverty that leads to the lower performance is regarded as “disadvantage,” how does this disadvantage in the primary effects turn around to become an “advantage” in the secondary effects? Most analyses in this regard have, as noticed above, tended to use a one-dimensional approach, with a three-way schema of parental class, and focus on contrasting performances between service- and working-class students and, in so doing, ignored ethnicity as a non-issue. Therefore, the questions that are of crucial importance for present research and that reflect the genuine concern of an increasingly diverse society were overlooked in most of the existing sociological analyses in this regard. As we take a multi-dimensional approach in the present study, we need to have a closer look at the other domains of socio-economic disadvantages that reinforce one another in their impact on ethnic minority students’ performance. Here the primary question we need to establish is: what kind of socio-economic disadvantages do members of ethnic minority heritages face?

Table 2 shows some selected family circumstances to represent social disadvantages: proportions of parents in working-class positions, with low level of or no formal education, of single-parent family type and being eligible for free school meal (FSM) which, for our sample, was equivalent to annual gross household income below £13,480 (Hobbs and Vignoles, 2010). These are, we believe, best available indicators of family economic, cultural and social deprivation.

**TABLE 2:** Selected family characteristics by ethnicity: proportions (%) growing up in working-class, poorly educated, single-parent households and being eligible for free school meal (FSM).

	Working-class	Low education	Single-parent HH	FSM
White	23	17	29	11
B Caribbean	32	20	64	18
B African	42	35	43	37
Indian	31	35	16	12
Pakistani	45	60	17	39
Bangladeshi	68	83	20	63
Chinese	31	56	12	5
Mixed	23	21	41	20
Other	36	45	28	33
(All)	25	20	29	13

It is clear that white students have much better socio-economic resources as judged from the range of indicators under consideration. White parents are least likely to be in work-class positions (23%) but ethnic minority parents are much more likely to be in such positions, with Pakistani and Bangladeshi parents particularly disadvantaged (68 and 45% respectively). Even more pronounced are differences in parental education, with only 17% of white parents having primary level or no schooling whereas for people from Bangladeshi, Pakistani and Chinese heritages, parental low education reaches a staggering high, at 83, 60 and 56% respectively. The combination of lower class position and poor education would mean that, even without labor market discrimination and differences in family size, ethnic minorities would have much greater vulnerability to poverty. While the large amount of missing income data in the NS file, at 58% as previously noted, makes it inadvisable to construct a poverty measure, we do have solid evidence on ethnic income poverty. Using the United Kingdom Household Longitudinal Study (UKHLS), Li (2018a: 487; see also Heath et al., 2018b) showed an ethnic poverty profile closely corresponding to the distributions to class and education position as shown in the table. The proportion of households in poverty, as defined by the United Kingdom government criteria (60% below median of the standardized household mean incomes) runs from 15, 21, 22, 25, 36, 49 to 56 percent for white, Indian, Black Caribbean, Chinese, Black African, Bangladeshi and Pakistani groups respectively. Although FSM eligibility does not fully reflect family poverty as Hobbs and Vignoles (2010) showed, we still find a close correspondence between indicators of socio-economic disadvantage (class, education, poverty) and FSM eligibility, with white students least likely and all other groups (except for Chinese) more likely to have FSM. Finally, single-parent family structure may be an indicator of inadequate family social capital crucial for the maintenance of cultural tradition (Sakamoto et al., 2009), “concerted acculturation” (Lareau, 2003) and emotional support (Putnam, 2007). Here we find that Black Caribbeans are most likely to live in single-parent families, with 64% being “always” or ‘sometimes’ headed by single parents, followed by Black Africans (43%).

Overall, data in Table 2 show that white students do enjoy superior socio-economic-cultural resources relative to their ethnic minority peers who face multiple disadvantages. People from Bangladeshi and Pakistani origins have the poorest economic situation, next come the Chinese in terms of low parental education, with the two black groups lying in between, and Indians being closest to whites. It is probably an interplay of these and other influences such as oriental cultural tradition (Hirschman and Wong, 1986) which emphasizes over-achievement and perception of pervasive disadvantages in the labor market such as shown in Li and Heath (2018), that led to the poorer academic performance but more ambitious choices for more advanced educational studies by the ethnic minority students. We now turn to multivariate modeling on such effects.

We first look at the net effects on academic performance as demonstrated in GCSE examination results. The data are shown in Table 3 with three models. Model 1 contains family class, ethnicity and gender, our key intersectional variables. Model 2 adds respondent-level FSM eligibility and school-level proportion of students eligible for FSM. The inclusion of the two FSM variables is of both conceptual and substantive importance. Conceptually, one may expect schools with high proportions of students eligible for FSM as being highly deprived and having an unfavourable learning environment, a negative effect over and above personal poverty (own FSM). Controlling for individual and school-level FSMs can hopefully help mitigate ecological and atomistic fallacies. Substantively, while Siddiqui et al. (2019) suggest that with the availability of individual FSM data, there is no need to include family circumstances such as parental class and education, we can directly test whether parental position is still significant after controlling for both individual- and school-level types of FSM. One further consideration is that Ilie et al. (2017) recommend using two other contextual-level deprivation indices in lieu of FSM, but our prior analysis suggests little need for so doing.^5^ The results in Model 2 can help us to address the questions of relative merits or otherwise of the claims from different theoretical perspectives as outlined above. Finally, Model 3 adds variables on parental education, family structure, nativity, and school-level ethnic diversity as measured by the Herfindahl index. Sociologists tend to use parental class alone as family position in addressing intergenerational educational or occupational mobility (Halsey et al., 1980; Goldthorpe and Jackson, 2008; Breen et al., 2009) but increasingly there is an appreciation that parental education plays a crucial role over and above parental class in shaping children’s educational and occupational attainment when parental education is used as a “positional good.” namely, in a relative rather than absolute sense (Bukodi et al., 2014; Li, 2018a). As the cohort members in the present study are of the same age, there is no need to produce relative measures of parental education. Finally, as students are nested in schools and as schools differ in the levels of socio-economic deprivation and ethnic diversity, multilevel regression techniques are used, with school-level FSM and Herfindahl diversity serving as level-2 covariates.

**TABLE 3:** Random coefficient models on GCSE scores by socio-economic attributes.

	Model 1	Model 2	Model 3
Parental class (routine = ref)			
Higher salariat	20.55***	17.79***	10.34***
Lower salariat	15.33***	12.57***	7.60***
Intermediate	10.95***	8.44***	6.09***
Own account	8.81***	6.04***	3.72***
Supervisor and technician	5.83***	3.80***	2.21**
Semi routine	4.64***	3.12***	2.66***
Ethnicity (white = ref)			
B Caribbean	−4.84***	−3.25**	−4.97***
B African	1.30	4.01***	0.03
Indian	6.52***	7.45***	6.00***
Pakistani	2.47**	5.15***	4.65***
Bangladeshi	6.32***	10.08***	10.36***
Chinese	16.97***	18.63***	19.08***
Mixed	−0.41	0.72	−0.19
Other	5.10***	7.05***	5.26**
Female	4.64***	4.71***	4.67***
Eligibility for FSM		−6.15***	−4.53***
% Eligible for FSM at school level		−0.37***	−0.41***
Parental education (low = ref)			
Degree^+^			15.13***
Sub-degree			8.40***
A-level			6.76***
O-level			4.39***
Family structure (two-parents = ref)			
Sometimes lone-parent			−2.02***
Always lone-parent			−2.32**
Born outside the United Kingdom			3.03***
Ethnic diversity at school level			0.10***
Constant	24.99***	33.70***	29.00***
Random effects parameters			
var (zfsmpct)	2.23***	1.77***	1.68***
var (zdiv)	−6.48**	−5.51	−10.07***
var (_cons)	1.86***	1.82***	1.70***
var (Residual)	2.76***	2.76***	2.73***
(N)	11,099	10,645	10,374

Note: *p < 0.05; **p < 0.01; ***p < 0.001. For parental education, low refers to primary level or no formal education. In the part for random effects, zfsmpct and zdiv refer to standardized values of percentage of pupils eligible for free school meals and of ethnic diversity at the school level respectively. Stata calculates the variances for the random parameters in the form of the log of standard deviations. The values of the logs are presented in the random part.

The data in Model 1 of Table 3 show powerful class and some ethnic and gender effects net of one another. Students from higher salariat families have, controlling for ethnicity and gender, 20.6 scores higher than those from routine families. We noticed in Table 1 that Pakistani and Bangladeshi students had lower mean GCSE scores than white students and, from Table 2, we also saw that their family class and education positions were much lower than those of whites. Yet, here, we find that their performance is significantly higher than that of white pupils, suggesting that it was their lower parental class that suppressed the achievement. With similar family positions, Pakistani and Bangladeshi students would perform equally well as, or probably better than, their white peers. Girls, on average, outperformed boys even when parental class and ethnicity are held constant.

As people eligible for FSM tend to be from poor households, we would expect them to have, other things being equal, lower levels of academic performance, which is shown as true. They have six scores lower on average. Furthermore, we find that school-level FSM also have a net and substantial impact on students’ performance. With an overall FSM at around 14%, an increase of ten percentage points of school-level FSM would, other things being equal, lower a student’s performance by around four scores. As most of the ethnic minority students except Indians and Chinese were more likely to be in receipt of FSM, controlling for individual and school level FSM have placed them on higher (net) performance scores than white students.

Finally, in Model 3, we find that parental education, family structure, nativity and school-level ethnic diversity all play an important role. People with degree-level parents have, other things being equal, 15 scores higher than those whose parents have only primary level of education or no formal schooling. People growing up in lone-parent families, whether “sometimes” or “always” lone-parent, also had lower scores. Yet, those who were foreign born but who arrived in the United Kingdom at a young age achieved higher scores than did the others, by three points on average, possibly reflecting the “positive selection” effect due to the recency of immigration and their parental higher qualifications.^6^ School-level ethnic diversity also has a positive impact on students’ achievement.

An interesting and important point is that, after controlling all these individual and contextual factors, we still find highly significant effects of parental class and ethnicity. Combining the findings from Tables 1–3, we may say that most ethnic minority students had lower performance scores due to the multiple handicaps arising from “inequalities of condition” inherent in their family position and, yet, if they had had comparable parental socio-economic conditions to those found in white families, they may well have obtained similar, or even better, results. Only Black Caribbean students might have fared worse.

### Transition to A-Level Studies

We now move to the choices made by the young people to follow A-Level studies. Most existing work on primary and secondary effects have focused on this, with the secondary effects gleaned from differences between salariat- and working-class children. Our analysis in Table 4 follows the structure of Table 3, with Model 1 focused on intersectional effects, Model 2 adding prior levels of achievement to assess secondary effects, and Model 3 further controlling for other individual and contextual factors. The data in Table 4 show average marginal effects (AME) from logit models, with logit coefficients transformed to proportions, or transition rates, to A-Level work.

**TABLE 4:** Average marginal effects (AME) from logit models on transition into A-Level work by socio-economic attributes.

	Model 1	Model 2	Model 3
Parental class (routine = ref)			
Higher salariat	0.454***	0.037	0.022
Lower salariat	0.331***	0.005	−0.009
Intermediate	0.230***	−0.012	−0.009
Own account	0.201***	−0.013	−0.010
Supervisor and technician	0.095***	−0.034	−0.031
Semi routine	0.093***	−0.001	−0.001
Ethnicity (white = ref)			
B Caribbean	−0.031	0.088**	0.068*
B African	0.154***	0.158***	0.107**
Indian	0.215***	0.149***	0.144***
Pakistani	0.165***	0.166***	0.147***
Bangladeshi	0.181***	0.145***	0.122***
Chinese	0.318***	0.142	0.139
Mixed	0.009	0.028	0.021
Other	0.175***	0.132***	0.107**
Female	0.071***	0.023*	0.028**
GCSE		0.013***	0.013***
Eligibility for FSM			0.025
% Eligible for FSM at school level			0.000
Parental education (prim = ref)			
Degree^+^			0.078***
Sub-degree			0.043*
A-level			−0.001
O-level			0.016
Family structure (two-parents = ref)			
Sometimes lone-parent			−0.006
Always lone-parent			−0.017
Born outside the United Kingdom			0.043
Ethnic diversity at school level			0.001
(N)	8,641	8,641	7,971

Note: *p < 0.05; **p < 0.01; ***p < 0.001. For parental education, low refers to primary level or no formal education.

The data in Model 1 shows the expected class differentials. Ethnic and gender status being equal, those from higher salariat families were 45 percentage points more likely to choose A-Level studies than those from routine families. Most people from ethnic minority backgrounds are also significantly more likely to choose A-Level studies than the white majority, holding constant family class position. As ethnic parents have lower class positions than whites, controlling for class boosted their transition rates as compared with the raw figures shown in Table 1. Girls are significantly more likely to choose A-Level studies than boys.

The crucial findings are shown in Models 2 and 3 where academic performance and other personal and contextual attributes are taken into account. It is surprising that parental class loses its significance altogether. Chinese students have very high GCSE scores, but once prior performance is controlled for, they are not significantly more likely to opt for A-Level studies. The overall pattern in Model 2 is echoed in Model 3 when the other factors are controlled for. The most salient feature that emerges from the findings under the two models is the lack of significant parental class effects. One reason for the difference in the findings as shown here and those by Goldthorpe and colleagues as cited above may be due to the number of class categories used: a seven-class schema is used here but a three-class schema used in their analyses; another reason may be due to the inclusion of ethnicity, gender and other covariates here, making the analysis more complicated, diluting the impacts of class. To further ascertain why the discrepancy emerged, further analysis was conducted, with a three-way schema for parental class, and with GCSE scores normalized with a mean of zero and standard deviation of unity, which is the same framework as adopted in prior analysis (Erikson et al., 2005; Jackson et al., 2007; Goldthorpe and Jackson, 2008; Jackson, 2012); Jackson, 2013.

**FIGURE 1:**
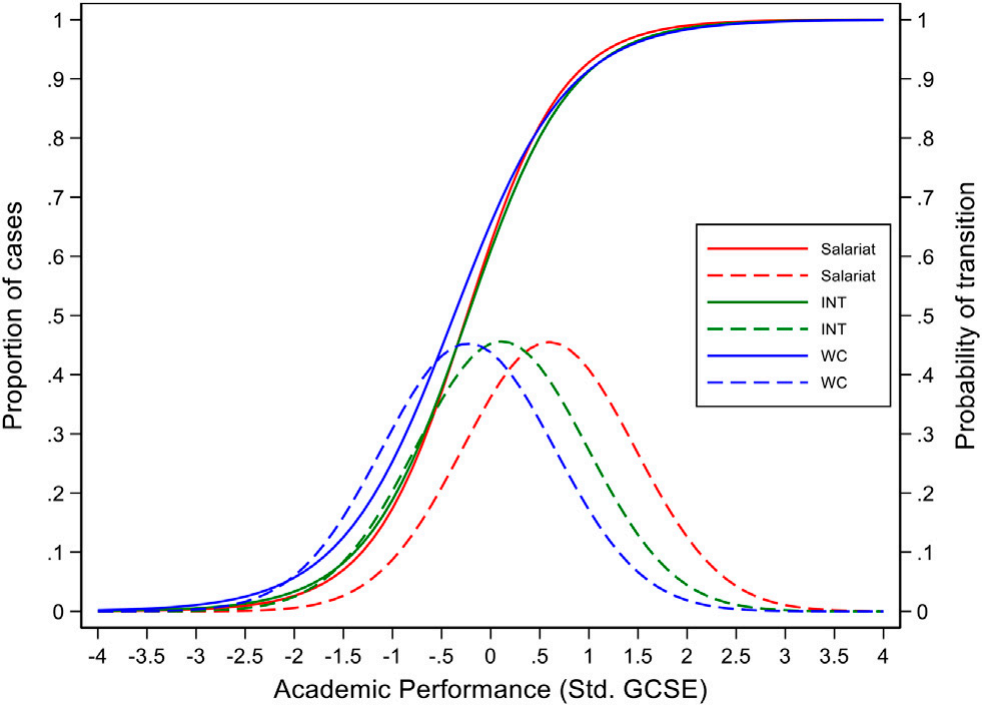
Graphical representation of regression of transition to A-level work on academic performance.

The data in Figure 1 shows clear class differences in the primary effects, with students from salariat families having much higher scores than those from working-class families, which closely resembles previous findings by other scholars using other datasets. Yet, controlling for prior attainment, the differences in the transition rates, or the secondary effects, for children from the three classes as shown in the S-shaped curves are quite indiscernible. Does this contradict the predictions of the rational action theory that middle-class children will tend to make more ambitious choices and working-class children more realistically-feasible choices? Probably not. If we compare the historical trends on transition rates between the NDCS (born in 1958 and reaching age 16 in 1974) and the 2001 YCS data as shown in Goldthorpe and Jackson (2008, **Figures 3.1** and **3.2**), we can see that the secondary effects were being reduced from earlier to later time points, suggesting that all children were becoming more likely to continue with A-Level studies. Our NS children’s transition time occurred in around 2006, even later than in the YCS2001 data, hence the class differences may be expected to be even smaller than shown in the YCS2001. From this perspective, we may say that even if primary effects remain, the strength of secondary effects may well decline or shift to more advanced levels, and this explanation would be consistent with Goldthorpe’s critique of Bourdieu’s cultural capital (habitus) theory, and with the “maximum maintained inequality” (MMI) and the “effectively maintained inequality” (EMI) theses by Raftery and Hout (1993); Lucas (2001).

Another feature in this regard that merits further consideration pertains to the possibility that the secondary effects may not cover the whole range of performance but only emerge at a particular performance level. Jackson et al. (2007: 218) state: “It would seem reasonable to suppose that students who perform very poorly in their examinations at 16 will have a low probability of going on to A-levels and that those who perform very well will have a high probability almost regardless of their class origins, while it is at intermediate levels of performance that the scope for secondary effects to operate is largest.” We can have a closer look to see whether this proposition is verifiable in our data.

The data in Table 5 are organized for this purpose. Academic performances (GCSE scores) are divided into three bands: low, middle and high. In the last row of the table, we find that the transition rates for A-Level studies under the three bands are 18, 56 and 93 percent. Thus those in the high band of achievement are around 5 times as likely to make the decision to go on to A-Level studies as those in the low band. Do we find class differentials only among the middle-band achievers but not among the high and the low achievers? Surprisingly, we do not. The first three rows under “All” show little class difference among the low and the mid, but significant class differences among the high, achievers. A very high proportion of high-achievers from all class origins choose to move to A-Level studies and working-class high-performers have a higher rate than salariat low- or mid-performers. But a close look still shows that, among the high performers, working- and intermediate-class children have a significantly lower rate than salariat children, at 86, 91, 94% respectively. Thus, our data show a pattern of secondary effects only among the high-achievers rather than among the intermediate performers as Jackson et al. (2007) have expected.

**TABLE 5:** Transition rate (%) into A-levels work by family class, ethnicity, sex and bands of GCSE scores.

	Bands of GCSE scores		
	Low	Mid	High
All			
Salariat (=ref)	20	57	94
Intermediate	16	53	91**
Working class	19	57	86***
White men			
Salariat (=ref)	17	53	94
Intermediate	11	48	90*
Working class	17	52	76***
White women			
Salariat (=ref)	18	59	94
Intermediate	15	49*	91
Working class	17	52	87**
Ethnic minority men			
Salariat (=ref)	26	67	96
Intermediate	29	77	91
Working class	26	72	95
Ethnic minority women			
Salariat (=ref)	52	72	97
Intermediate	43	82	95
Working class	30*	75	94
(All)	18	56	93

Note: The figures in this table pertain to the percentages that transition into A-Level studies. Further analysis is made on significance tests with people from salariat origins as the reference group. For instance, at the overall level (under All), 86% of people from working-class origins as against 94% of salariat children made the transition to A-Level studies, with a difference of 8 percentage points, and this is significant at the 0.001 level.

Since we are also concerned with ethno-gender differences, further analysis is conducted on ethno-class-gender effects on children’s performance and transition probabilities, with results listed in the lower part of the table. Here we find that the RRA predictions mainly apply to the high-achieving white students. For both men and women in the majority group, there are clear and significant class differences among the high achievers. For ethnic minorities, however, it is academic performance rather than parental class position that plays a more decisive role. It is noted here that even at the low level of performance, ethnic men and women are more likely to make the transition than their white peers. Yet it is also the case that among ethnic minority women in the low band, class differences exist, with working-class girls being 22 percentage points behind their salariat counterparts in the transition rates (30 and 52% respectively), which constitutes a statistically significant difference. Further analysis shows that all low-performing working-class girls from ethnic minority heritages apart from Black Africans (no Chinese girls were in this category) had low transition rates, at 30, 26, 22, 29 percent for Black Caribbean, Indian, Pakistani and Bangladeshi groups although they were still more likely to opt for A-Level studies than their white counterparts from salariat families.

Overall, our analysis has enhanced the application of the rational action theory with regard to the class-ethno-gender specificity rather than showing encompassing support. With this mind, we move on to the transition to university including Russell Group universities.

### Transition to University

Table 6 shows the transition rates to university (Models 1 and 2) and to Russell Group (RG) universities. Models 1 and 3 show the intersectional effects and Models 2 and 4 show full effects akin to Model 3 in Table 4. The data in Model 1 on access to university are similar to those in Model 1 on transition to A-Level studies, showing pronounced class and clear ethno-gender effects. The only notable differences between the patterns shown here and those revealed previously on transition to A-Level studies are that family class and ethnicity effects are even more pronounced here on access to university, suggesting that the higher the level of educational attendance, the more important the family class position and that white working-class children are being left further behind. With regard to the secondary effects, we need to take into account prior performance but there is no clear guidance as to what can effectively serve as such an indicator: one could use GCSE scores, number of A-C grades, or having achieved five or more A-C grades at GCSE or equivalent including English and Mathematics. After some careful comparison, we decided to adopt the last of these as it is an important and quite commonly used indicator. 51% of white as compared with 35% Black Caribbean and 39% of Pakistani students achieved this, with Chinese (78%) and Indians (61%) being in the lead, and Black African (45%) and Bangladeshi (43%) students being in the middle. In addition, the other personal and contextual variables as previously used are included in the model for as covariates.

**TABLE 6:** Average marginal effects (AME) from logit models on access to university and to Russell-Group (RG) universities.

	Access to university		Access to RG universities	
	Model 1	Model 2	Model 3	Model 4
Parental class (routine = ref)				
Higher salariat	0.528***	0.144***	0.246***	0.044**
Lower salariat	0.357***	0.100***	0.099***	0.006
Intermediate	0.224***	0.069**	0.053***	0.001
Own account	0.187***	0.047*	0.035***	−0.008
Supervisor and technician	0.109***	0.024	0.011	−0.012
Semi routine	0.077***	0.042	0.013*	0.001
Ethnicity (white = ref)				
B Caribbean	0.031	0.100**	−0.069***	−0.042**
B African	0.309***	0.258***	−0.002	0.002
Indian	0.394***	0.326***	0.051**	0.031*
Pakistani	0.235***	0.249***	0.020	0.029
Bangladeshi	0.331***	0.337***	0.092*	0.109**
Chinese	0.598***	0.588***	0.235*	0.111
Mixed	0.029	0.023	0.005	−0.005
Other	0.207***	0.184***	0.041	−0.017
Female	0.077***	0.048***	0.018**	0.003
Eligible for FSM		−0.015		0.006
% FSM in school		−0.002***		−0.002***
Parental education (low = ref)				
Degree^+^		0.150***		0.069***
Sub-degree		0.084***		0.002
A-level		0.023		0.001
O-level		0.019		−0.023*
Fam structure (2 parents = ref)				
Sometimes lone-parent		−0.026*		−0.017*
Always lone-parent		−0.014		0.020
Born outside the United Kingdom		0.066**		0.032*
Ethnic diversity at school		0.001**		0.001**
Five A-C incl E&M		0.352***		0.203***
Pseudo R^2^	0.112	0.307	0.099	0.264
(N)	8,105	7,489	8,105	7,489

Notes: *p < 0.05; **p < 0.01; ***p < 0.001. “Five A-C incl E&M” refers to ‘Achieved 5 or more GCSEs or equivalent at A*-C grade including English and Maths.

The data in Model 2 shows that achieving five or more GCSE A-C grades including English and Mathematics is of crucial importance in securing a place in university. Other things being equal, those students with this level of achievement have a transition rate being 35 percentage points higher than those without this attainment. Parental education has a positive effect but coming from single-parent family has a negative effect. School-level poverty (in terms of percentage FSM eligibility) and ethnic diversity have the effect as expected. Controlling for these, we find that ethnic effects were little changed but class effects declined sharply. Yet, these declines notwithstanding, it is still the case that those from salariat families are more likely to be enrolled in university by around 10–15 percentage points, and those from intermediate families by around five points, than working-class students. The class advantage as shown here echoes what Goldthorpe and colleagues observed for transitions to A-Level study, and the pattern again renders support to relative risk aversion thesis.

The main features of access to university are largely echoed in access to Russell Group universities, albeit with weaker strengths due to the small numbers involved. As Bangladeshi students tend to face more disadvantages in terms of parental class and primary attainment, they are found to have a higher probability of accessing Russell Group universities when prior conditions are held constant, in contrast to Black Caribbean students.

### Labor Market Position

Having looked at the educational trajectory in some detail, we move to the respondents’ labor market situation in wave 8 when they were aged 25. In the preceding analysis, we found that ethnic minority students, with the exception of Chinese and Indians, performed less well than did white students in the primary effects but better in the second effects. The first result arose chiefly from family disadvantages and the second result obtained in spite of family poverty. A question that would lend itself in this regard is: did their aspiration, determination and efforts pay off? In other words, did ethnic minority students obtain occupational and earnings’ positon commensurate with their human capital investment? How well did they fare in their earlier career life as compared with their white peers?

Table 7 shows the main characteristics of the respondents’ human capital and labor market positions at wave 8. The data cover percentage with a degree, labour-market position, and gross and net weekly incomes by ethnicity.^7^ Labor market position is a combination of employment status and class position with four categories: salariat and non-salariat among the employed, and unemployed and inactive among the workless. Gross weekly pay is payment from the main job for those in employment, with the workless including the unemployed. full-time students, looking after home and sick and disabled having no earnings from the labor market. 36 of the respondents reported abnormally high earnings (over £100 per hour) and these are omitted from analysis following the government instructions in the collection of earnings data (see Labour Force Survey, 2015: 384). It is clear that people of ethnic minority heritages are well educated and have a higher likelihood of having a degree-level qualification than do the majority, with those from Black African, Indian and Chinese heritages having a probability nearly twice as high. It is noteworthy in this regard that even those from Pakistani, Bangladeshi and Black Caribbean origins who grew up in poverty-ridden homes outperform whites in gaining a degree qualification.

**TABLE 7:** Education, labor market position and income (£) by ethnicity (N = 7,707).

	% degree	Labor market position (%)				Gross pay (£)	Weekly income (£)
		Salariat	Other	Unem	Inactive		
White	25	35	48	6	10	351	313
B Caribbean	28	25	58	11	7	270	227
B African	50	45	39	12	5	338	228
Indian	49	50	37	10	3	362	246
Pakistani	31	34	43	11	11	263	217
Bangladeshi	27	37	44	8	11	321	213
Chinese	45	54	36	9	0	394	224
Mixed	29	34	48	10	8	322	236
Other	47	37	43	13	6	339	227
(All)	27	36	48	7	9	347	300

Note: Full-time students are omitted in analysis of the labor market position. “Other” refers to those in non-salariat’ jobs, and ‘Unem’ to the unemployed. Gross pay refers to gross weekly earnings from the main job but excludes the small number of respondents (N = 36) with abnormally high pay (over £100 per hour) in accordance with government instructions on collection of earnings data. Continuous weekly income pertains to take-home income for cohort member and partner as derived from banded incomes (1 = under 25 … 16 = more than 1,400).

With such a high educational profile, we would have reason to expect ethnic minority groups to make similarly impressive progress in the labor market positions. Unlikely their parents, they do not have language problems and their social capital is similar to that of white students. Yet, when we turn our gaze to employment and income situation, we are disappointed. The educational attainment by the ethnic minorities did not have the returns as expected. Every minority group were more likely to be unemployed, with the two black groups and Pakistanis being nearly twice as likely as whites to face unemployment, and that in spite of the higher educational qualifications. For those lucky enough to have a job, the chances of securing a “nice” job (in professional-managerial salariat position) are not too bad, although they may still be regarded as being disadvantaged if educational attainment is taken into account. For instance, 50% of Black Africans and 25% of whites had degree-level education but the salariat occupancy of the former is only slightly higher than that of the latter (45 vs. 35%). What is of even greater concern is the fact that, despite the higher levels of educational qualifications and of somewhat similar levels of occupational attainment (for those with a job), the two black groups and the two Muslim groups (Pakistani and Bangladeshi) have notably lower gross weekly earnings, and the “continuous weekly income” for the cohort member and partner is much lower for all ethnic minorities than for whites, suggesting lower returns to education and labor market position and greater economic disadvantages for the ethnic minorities.

Finally, we take a look at the two kinds of income data: gross weekly earnings and continuous weekly income. For the former, we use the Heckman regression method as the earnings depend on being employed. For the selection part, we use limiting long-term illness as the “identifying” variable in addition to other variables that are also used in the regression part. As the probit coefficients predicting whether earnings’ data are actually observed are not intuitive, we have transformed into percentages using the average marginal effects. Thus the first two columns in Table 8 refer to the avoidance of worklessness and the last two columns to the earnings differentials conditional on employment. Under both selection and regression parts, we use two models. Model 1 includes family class, ethnicity and gender, and Model 2 includes marital status, number of dependent children, and parental and own education.

**TABLE 8:** Average marginal effects (AME) on avoidance of worklessness (%) and gross weekly earnings (£) conditional on employment based on Heckman’s model.

	Avoidance of worklessness (%)		Growth weekly earnings (£) conditional on employment	
Parental class (routine = ref)				
Higher salariat	0.264***	0.108**	161.80***	79.87***
Lower salariat	0.223***	0.097**	103.89***	41.86
Intermediate	0.177***	0.066	83.43***	32.59
Own account	0.138***	0.050	70.71***	28.76
Supervisor and technician	0.136***	0.081*	39.86*	21.05
Semi routine	0.077*	0.060	21.20	3.09
Ethnicity (white = ref)				
B Caribbean	−0.090*	−0.071	−52.56**	−50.54***
B African	−0.083	−0.176***	49.34*	0.35
Indian	−0.056	−0.152***	66.89***	31.38*
Pakistani	−0.104**	−0.139***	−2.87	−24.62
Bangladeshi	−0.061	−0.113**	22.88	−9.63
Chinese	−0.159	−0.132	125.89*	76.11
Mixed	−0.060	−0.070	−0.17	−3.59
Other	−0.129*	−0.171**	70.12	38.15
Female	−0.003	−0.001	−88.23***	−96.15***
Marital status (single = ref)				
Married		0.013		33.61**
Divorced/separated		−0.090		−58.90**
Number of children in HH		−0.069***		−32.73***
Parental education (low = ref)				
Degree+		0.022		41.08**
Sub-degree		0.020		22.46
A-level		0.063**		18.58
O-level		0.075***		22.21
R’s education (low = ref)				
Degree+		0.264***		124.98***
Sub-degree		0.169***		70.99***
A-level		0.231***		92.72***
O-level		0.137***		53.38***
Limiting LT illness	−0.152***	−0.130***		
N	6,703	6,265	6,703	6,265

Note: Full-time students at Wave 8 were omitted from analysis.

Looking firstly at the joint effects of worklessness in the selection part, we find that parental class exerts a powerful influence, with those from higher salariat families being 26.4 percentage points more likely to be in employment than those from routine manual families, other things being equal, with clear class gradients. Holding constant family class, all ethnic minority groups were less likely to be in employment, with Black Caribbean and Pakistani respondents being nine and ten percentage points less likely than whites to be employed. Under Model 2 when the other covariates are taken into account, we find, as expected, highly salient effects of own education and fairly noticeable parental educational effects, but parental class effects are much reduced. Yet, interestingly, controlling for education brought the ethnic penalties into much sharper relief, with those of Black African, Indian, Pakistani and Bangladeshi heritages being significantly more likely to face worklessness than whites, and the magnitude ranged between 11 and 18 percentage points higher.^8^

For those fortunate enough to be in employment, family class still plays a highly important role, and Black Caribbeans and female respondents receive much less gross weekly pay, with Indians and Chinese having significantly more gross weekly earnings. When the other factors are taken into account, family class effects are sharply reduced. Black Caribbean’s penalty remains at a similar level although Indians’ and Chinese premiums are much reduced. People’s own education plays a very important role. Demographic attributes like gender, marital status and number of dependent children play a more salient role in terms of the amount of earnings than the probability of being in employment, other things being equal.^9^

As around 12 percent of the respondents are married or partnered^10^ who are expected to share economic weal and woe, and as those not in employment may have other sources of income, we now turn to the “continuous weekly income,” that is, incomes from all sources, which is a good measure of the overall economic well-being of our respondents. The data, obtained from OLS analysis, are shown in Table 9 with four models. Model 1 contains our main variables on parental class, ethnicity and gender, Model 2 adds personal attributes on marital status, number of children and health condition (in terms of GHQ12),^11^ Model 3 further adds parental and own education and, finally in Model 4, we add respondents’ own class position differentiating salariat, non-salariat and workless.

**TABLE 9:** OLS regression of weakly take-home income (£).

	Model 1	Model 2	Model 3	Model 4
Parental class (routine = ref)				
Higher salariat	102.79***	81.61***	52.05***	51.53***
Lower salariat	88.30***	72.82***	46.47***	45.58***
Intermediate	74.27***	62.31***	41.12***	40.93***
Own account	64.58***	51.41***	35.74***	35.10***
Supervisor and technician	53.25***	41.60***	30.18***	29.29***
Semi routine	16.85***	11.97***	4.90	4.55
Ethnicity (white = ref)				
B Caribbean	−81.27***	−91.36***	−89.65***	−88.95***
B African	−76.33***	−88.68***	−94.67***	−94.78***
Indian	−55.56***	−69.23***	−70.29***	−70.53***
Pakistani	−70.01***	−85.27***	−79.46***	−78.98***
Bangladeshi	−58.88***	−80.89***	−70.51***	−70.50***
Chinese	−77.49***	−84.45***	−80.68***	−79.62***
Mixed	−77.49***	−83.62***	−83.92***	−83.73***
Other	−76.58***	−85.80***	−86.86***	−88.30***
Female	32.09***	31.11***	27.58***	27.85***
Marital status (single = ref)				
Married		6.79***	4.63*	4.97**
Divorced/separated		−5.90	−2.21	−2.72
Number of children in HH		−12.29***	−4.87***	−2.92**
Health status (GHQ12)		−0.66***	−0.57**	−0.36
Parental education (low = ref)				
Degree+			36.76***	35.42***
Sub-degree			38.19***	36.53***
A-level			34.53***	33.50***
O-level			33.55***	32.76***
R’s education (low = ref)				
Degree+			41.95***	37.13***
Sub-degree			29.35***	26.32***
A-level			35.53***	32.34***
O-level			20.83***	19.00***
LM position (workless = ref)				
Salariat				17.26***
Non-salariat				9.47***
Constant	232.88***	263.59***	224.81***	216.56***
R^2^	0.413	0.523	0.592	0.596
N	7,231	6,913	6,473	6,106

The data in Table 9 show marked ethnic disadvantages. Firstly, we find that, under Model 1, parental class exerts a huge impact on people’s income, with those from higher salariat families having over £100 per week than those from routine families, a difference similar to that found by Laurison and Friedman (2016). After taking parental class into consideration, we find that ethnic minorities have much lower incomes, ranging from 56 to 81 pounds less than whites. As ethnic minorities’ parental class are generally in low positions, controlling for parental class makes little impact on respondents’ income differentials, which is clearly shown when we compare the findings under model 1 with those under the last column of Table 7. As our respondents were still young in wave 8, most of them were unmarried and only a small portion of them had children or health issues, controlling for these factors does not change the patterns very much. In model 3 where we further control for parental and own education, we find that educational qualifications make a big difference and that, as a result, parental class effect is almost halved. In model 4, we further control for respondents’ own class position. Here we find that, as expected, people in salariat positions have higher weekly incomes than do the workless (unemployed + inactive). Yet, it is also important to note that, if we compare the figures from models 1–4, we find that, as more variables are controlled for, parental class effects are progressively reduced whereas ethnic effects are actually increased. For instance, respondents from higher salariat families are found to have £102.8 more weekly income in model 1 than do those from routine families, holding constant ethnicity and gender effects, but when the other factors are taken into account in model 4, the class differential is reduced to £51.5. If we look at Black Africans’ income, we find that they have, given parental class and gender status, £76.3 less per week in model 1 than do white respondents but when all other factors are taken into account in model 4, their income differentials becomes larger, at £94.8 less. People prefer to “compare like with like,” but the more like the personal and other characteristics we compare, the more unlike the take-home incomes between the ethnic minority and the majority groups we find.

## Discussion and Conclusion

This paper has sought to contribute to scholarship on socio-ethno differences in British society. Most existing analyses on primary and secondary effects have confined their efforts to a three-way parental class effects on GCSE scores and transition to A-Level studies. Using the Longitudinal Study of Young Persons in England (LSYPE1, also known as Next Steps, NS), the present study has used a more elaborated seven-class NSSEC schema, and addressed class, ethnicity and gender effects simultaneously whilst controlling for parental education, family structure, economic situation (in terms of FSM eligibility) and contextual (school) level ethnic diversity and deprivation. We analyzed the socio-ethno differences not only in the primary and secondary effects during compulsory schooling, but in transition to university and to elite Russell Group universities too; and, furthermore, we linked the educational trajectory to labor market position and income profiles at age 25. Previous analyses in this area tend to focus on one or another specific aspect (Strand, 2007; Anders, 2012; Croll and Attwood, 2013; Anders, 2017; Ilie et al., 2017; Siddiqui et al., 2019; and those by Goldthorpe and his colleagues as noted above), but the present study has sought to provide a more systematic and comprehensive perspective.

The main findings can be summarized as follows. Firstly, there are pronounced parental class effects in all aspects under investigation: ranging from GCSE scores, transition rates to A-Level, university and elite (Russell Group) university studies, obtaining degrees, avoidance of worklessness to gross weekly earnings and continuous weekly take-home income. As ethnic minority groups come from disadvantaged families in terms of parental class, education and incomes, they tend to perform less well in school but are more likely to opt for A-Level and higher education studies, providing further evidence to the validity of the thesis of “reinvigorated aspirations” (Li, 2018a). Their attendance at elite universities is, on the whole, still lower than that of the white students, echoing previous findings by Boliver (2013).

The mainstream sociological analyses on primary and secondary effects have focused on parental class differences in academic performance at GCSE, and in transition rates to A-Level studies conditional on prior attainment. With respect to the secondary effects, the rational action theory expects the parental class effects to manifest themselves at lower levels of achievement or, more specifically, at the intermediate level. Most research in this respect has adopted a three-way class and ignored ethnicity and other factors. The present analysis has adopted a framework with a more elaborate class schema, with more explanatory variables and a greater coverage of analytical scope. Our analysis is not limited to testing the validity of the rational action theory concerning primary and secondary effects although we did find some support for the theory. Our findings in this regard are both substantively grounded and culturally fine-tuned.

The determination, ambition and aspiration of the young people from ethnic minority heritages were clearly shown in the choices they made with respect to transition to higher education. All members of ethnic minority groups were more likely to attend university and to hold a degree at age 25 that whites. Only Black Caribbeans were significantly less likely to attend elite Russell Groups universities.

All this suggests, as Li and Heath (2016) posit, a generally level playing ground of the educational system in Britain. Where ethnic minorities lag behind, such as in GCSE performance, it is mainly due to inequality of condition such as family and school deprivation rather than inequality of opportunity. They made laudable efforts in spite of family hardships, aimed higher and attained better educational qualifications. Given this, we might expect them to fare at least equally well in the labor market. Yet, to our dismay, we found that in spite of their better qualifications, they were more likely to face unemployment and inactivity, and had markedly lower weekly incomes even though among those lucky enough to be in employment, they were not too much disadvantaged (only Black Caribbeans were making significantly lower earnings). They started lower, worked harder, achieved well in education but were not fully rewarded in the labor market.^12^

Overall, we found persisting class effects and entrenched ethnic inequalities in British society. The first-generation immigrants may have been positively selected but they had to face the harsh reality in the labor market upon arrival in the United Kingdom, resulting in having depressed class positions and economic hardships. They may have passed their aspiration, determination and resilience to their children who, as we have seen, started from pervasive family poverty but made determined efforts at decision points, and achieved remarkable progress in educational attainment. Yet, in spite of all this, they still found themselves in greater worklessness resulting in lower incomes. The former Prime Minister Therese May (2017) said that continued ethnic disadvantages must be “explained or changed.” The analysis in this paper has sought to explain the entrenched ethnic disadvantages in British society, and our evidence calls for greater efforts by policy-makers, employers and wider society to adopt more decisive and more effective measures that can eliminate labour market discrimination against ethnic minorities, for social justice and for national prosperity.

## Data Availability Statement

Publicly available datasets were analyzed in this study. This data can be found here: discover.ukdataservice.ac.uk/series/?sn=2000030.

## Author Contributions

The author confirms being the sole contributor of this work and has approved it for publication.

## Conflict of Interest

The author declares that the research was conducted in the absence of any commercial or financial relationships that could be construed as a potential conflict of interest.

## Footnotes

1 Further analysis using the same data as Breen and colleagues used shows that if one focuses on degree-level education, the differences between children from the service class and those from the manual working-class families enlarged from 12.9 to 21.6 percentage points from the oldest to the youngest cohort, which would lend support to the declining mobility thesis by Blanden and her colleagues (2005). The two aspects are not in contradiction: a reduction of class differences at the lower levels of education was going hand in hand with an increase of class differences at the higher (degree or above) levels of education. As more people were attending the lower levels of education, this would lead to an overall reduction in educational inequalities but this does not prevent a deepening of class differences at the higher levels of education.

2 It is generally recognized that immigrants are positively selected in that they tend to have higher levels of education than do their fellow citizens in the country of origin. It would have been a nice idea to test whether our ethnic minority respondents’ parents were positively selected and, if so, by how much. In order to do this, we need information on their parents’ country of birth and time of arrival to the United Kingdom, but neither variable was available in the datasets. Even if the variables were available, it would still be impossible to test this idea fully because there is no information on the average education in all the countries and for all the years concerning immigrants to the United Kingdom. The Understanding Society (USoc) has data on father’s and mother’s country of birth (pacob macob) and respondents’ year of arrival to the United Kingdom (yr2uk4). In the 10 waves of the USoc, 148,337 people were interviewed, including 3,704 Indians. Excluding those with missing information on pacob and yr2uk4, there are 2,227 Indians in the file. From 1952 to 2017, there were Indians coming to the United Kingdom every year and their father’s country of birth includes India, Pakistan, Bangladesh, Italy, Sri Lanka, Kenya, Uganda and South Africa, Jamaica and other countries. But even using the best education data source currently available (http://www.barrolee.com/) would fail to provide the relevant information in most of the time-year-country combinations for Indians, let alone for all other immigrant groups. For instance, there was no information on average education in China before the 1980s (see Barro and Lee, 2010; Barro and Lee, 2013: 197) although Chinese immigrants started to arrive to the United Kingdom from 1946 in the USoc file. I wish to thank one of the reviewers for alerting me to this potentiality although I have been thinking about how to improve on this for years. Perhaps a better approach is to compare the immigrant’s parental class in the origin country with the white’s parental class in the United Kingdom. If immigrant fathers’ class position is similar to white fathers’ class, we would have reasons to believe that they are positively selected, as they tend to come from poor countries with low levels of socio-economic development. Research in this respect does support this idea Li, 2020).

3 Prior analysis showed little difference between “routine” and “never worked and long-term unemployed” categories in parental class, thus the two categories were combined to produce a seven-way parental class variable.

4 There are also data on access to Oxford and Cambridge Universities. Further analysis shows that 3.76% of respondents from higher salariat families attended these universities as compared with 0.22% from routine families, a disparity ratio of 17.

5 The two contextual variables recommended by Ilie et al. (2017) are idaci (“income deprivation affecting children index”) and imd (“index of multiple deprivation”). Analyses were conducted including the two variables, rescaled to range from 0 to 100, on top of the variables already in Model 3. The coefficients were rather weak: 0.08 and 0.02 respectively, the latter being non-significant and the coefficients for the other variables in Model 3 being little affected. Given this, the two variables were not included in the model.

6 Further analysis shows that around 5% of the sample were foreign born and came to the United Kingdom as children. Yet, among the foreign born, parental education is more stratified, with 23.8% of parents having degrees or higher, as compared with 18.4% of the United Kingdom born; yet the proportions having only primary or no education were also higher among the foreign born than the United Kingdom born, at 43.4 and 19.1% respectively. The positive selection effect is particularly strong among foreign born Chinese and white parents with 45 and 32% having degree-level education.

7 For brevity, we do not present parental raw class effects on respondent’s education, class and incomes here but will include the effects in the modeling. We have conducted the analysis and found salient effects in each of the domains. For instance, 44% of higher salariat children had degrees as compared with 13% from routine families. Similarly, 53% of the former held salariat positions as compared with 17% of the latter, and differences in “continuous weekly income” amounted to £108 (£343 for the former versus £235 for the latter).

8 An important question in this respect is whether ethnic minorities have equal returns to education in terms of employment opportunities, hence having earnings. Further analysis shows that at the degree level, the two black groups, Indians and Pakistanis were significantly less likely than whites to have a job; at the sub-degree level, Chinese were significantly behind whites; at A-Levels, the three South Asian groups were significantly behind; at the O-Levels, Chinese were significantly behind; and for those with only primary or no formal qualifications, Indians and Chinese are significantly behind. These findings are obtained with all other factors in the models held constant.

9 Again, a relevant question that poses itself is whether there are equal returns of education to earnings. Here, the significant effects are as follows: at the degree level, whites have £46 more than Black Caribbeans; at the sub-degree level whites have £83 and £286 more than Indians and Chinese respectively; at the A-Levels, whites have £93 more than Black Caribbeans, but £167 and £196 less than Indians and Chinese respectively; and at the O-Levels, whites make £70 and £218 more than Pakistani and Chinese respondents respectively, holding constant all other factors in the models.

10 The percentages of respondents who are married or partnered at the age of 25 are 11, 14, 30 and 25 for Whites, Indians, Pakistanis and Bangladeshis respectively. Seven percent of the mixed, six percent of Black Africans are also married. Only two percent of the Black Caribbeans and no one from the Chinese origins are found married.

11 Using information of “limiting long-term illness” does not change the main patterns of the other variables.

12 Even at age 25, 4.5% of the sample were still in education, with Chinese women and Black African men and women being much more so than others, at 29, 12, 12 percent respectively.
